# The role of neuroinflammation in cerebral amyloid angiopathy

**DOI:** 10.1016/j.ebiom.2024.105466

**Published:** 2024-11-29

**Authors:** Hilde van den Brink, Sabine Voigt, Mariel Kozberg, Ellis S. van Etten

**Affiliations:** a J. Philip Kistler Stroke Research Center, Department of Neurology, Massachusetts General Hospital, Harvard Medical School, Boston, MA, USA; b Department of Neurology, Leiden University Medical Centre, Leiden, the Netherlands; c Department of Radiology, Leiden University Medical Centre, Leiden, the Netherlands; d Department of Neurology, Massachusetts General Hospital, Harvard Medical School, Boston, MA, USA

**Keywords:** Cerebral amyloid angiopathy, Cerebral amyloid angiopathy-related inflammation, Neuroinflammation, Amyloid-β, ARIA

## Abstract

Cerebral amyloid angiopathy (CAA) is a cerebrovascular disease characterized by vascular amyloid-β (Aβ) deposition. CAA is often seen in the brains of elderly individuals and in a majority of patients with Alzheimer's disease. The molecular pathways triggered by vascular Aβ, causing vessel wall breakdown and ultimately leading to intracerebral haemorrhage and cognitive decline, remain poorly understood. The occurrence of CAA-related inflammation (CAA-ri) and Amyloid-Related Imaging Abnormalities (ARIA) have sparked interest for a role of neuroinflammation in CAA pathogenesis. This review discusses prior studies of neuroinflammation in CAA and outlines potential future research directions targeting candidates such as matrix metalloproteinases, complement activation, microglial activation, reactive astrocytes and parenchymal border macrophages. Understanding the role of neuroinflammation in CAA pathogenesis could help identify new therapeutic strategies.

**Search strategy and selection criteria**

Data for this Review were identified by searches—performed on January 31, 2024–of PubMed, Embase, Web of Science and Cochrane and references from relevant articles using the search terms ((“Cerebral Amyloid Angiopathy” [majr] OR “cerebral amyloid angiopathy” [ti] OR “cerebral amyloid angiopathies” [ti] OR “cerebral amyloid angiopathy” [title:∼3] OR “cerebral amyloid angiopathies” [title:∼3] OR “Cerebral Amyloid Angiopathy, Familial” [majr] OR “Autosomal Dominant Cerebrovascular Amyloidosis” [ti] OR “HCHWA” [ti] OR “hchwad” [ti] OR “Hereditary Cerebral Haemorrhage With Amyloidosis” [ti] OR “Hereditary Cerebral Haemorrhage With Amyloidosis” [ti] OR “Hereditary Cerebral Haemorrhage With Amyloidosis” [ti] OR “Hereditary Cerebral Haemorrhage With Amyloidosis” [ti] OR “Autosomal Dominant Cerebrovascular Amyloidosis” [title:∼3] OR “Icelandic Type Amyloidosis” [title:∼3] OR “Icelandic Type Cerebroarterial Amyloidosis” [title:∼3] OR “Hereditary Cerebral Haemorrhage With Amyloidosis” [title:∼3] OR “Hereditary Cerebral Haemorrhage With Amyloidosis” [title:∼3] OR “Hereditary Cerebral Haemorrhage With Amyloidosis” [title:∼3] OR “Hereditary Cerebral Haemorrhage With Amyloidosis” [title:∼3] OR ((“CAA” [ti] OR “dcaa” [ti] OR “d caa” [ti]) AND (“angiopathy” [ti] OR “angiopathies” [ti] OR “amyloid” [ti] OR “amyloid∗” [ti])) OR (“cerebral” [ti] AND “amyloid angiopath∗” [ti])) AND (“Inflammation” [majr] OR “Inflammation” [ti] OR “Inflammatory” [ti] OR “Inflammat∗” [ti] OR “Neuroinflammation” [ti] OR “Neuroinflammatory” [ti] OR “Neuroinflammat∗” [ti] OR “Complement System Proteins” [majr] OR “Complement Activation” [majr] OR “Complement Activation” [ti] OR “Complement Pathway” [ti] OR “Complement Pathways” [ti] OR “Complement system” [ti] OR “Complement Cascade” [ti] OR “Complement Component” [ti] OR “Complement Components” [ti] OR “Receptors, Complement” [majr] OR “Complement Receptor” [ti] OR “Complement receptors” [ti]) AND (“2000/01/01” [PDAT]: “3000/12/31” [PDAT])). Only original research papers published in English were reviewed. The final reference list was selected based on originality and relevance to the scope of this review.

## Introduction

The global rise in life expectancy predicts a surge in age-related cerebral small vessel diseases, including cerebral amyloid angiopathy (CAA). Characterized histopathologically by amyloid-beta (Aβ) accumulation in vessel walls, CAA affects approximately 25% of the elderly and coexists with Alzheimer's disease (AD) in at least 50% of cases.^1^ Clinical presentations include symptomatic lobar intracerebral haemorrhage, cognitive decline, and transient focal neurological episodes.^2^ Additional haemorrhagic CAA lesions include cerebral microbleeds, which represent the rupture of individual cortical arterioles,^3^ and cortical superficial siderosis, a chronic manifestation of convexity subarachnoid haemorrhage and a strong predictor of lobar haemorrhage. Non-haemorrhagic brain injuries have also been associated with CAA, including white matter hyperintensities and microinfarcts, and these lesions correlate with cognitive impairment.^2^ Recent studies have shown evidence that neuroinflammation might play a role in the steps between vascular Aβ accumulation and vascular injury resulting in vessel rupture and haemorrhage or ischemic damage to the brain. Of note, various lines of evidence indicate that the activation of immune mediators plays a crucial role in regulating AD pathology with prominent pathological features observed in the AD brain including reactive astrogliosis and microgliosis.^4^ However, the precise role of neuroinflammation – and whether it is an epiphenomenon or a causal factor in vascular damage-remains less clear.

One line of evidence supporting the role of neuroinflammation in CAA comes from the emergence of patients with CAA that experience CAA-related inflammation (CAA-ri), a condition in which perivascular and intravascular inflammation related to vascular Aβ leads to local blood–brain barrier leakage and vessel rupture. Additionally, CAA has been linked to adverse reactions in patients receiving novel anti-amyloid immunotherapies for AD, known as Amyloid-Related Imaging Abnormalities (ARIA),^2^ which has a notably similar clinical and radiographic presentation to CAA-ri. Besides the clinical syndromes of CAA-ri and ARIA, Aβ in itself can trigger sub-clinical chronic inflammation in the form of activated microglia and pro-inflammatory agents,^5^ potentially exacerbating vascular damage in all patients with CAA. Interestingly, vascular Aβ is often absent at vessel rupture sites, suggesting that local inflammation may remove Aβ from the vessels before bleeding occurs.^3^^,^^6^

This review integrates evidence concerning the involvement of neuroinflammation in CAA-related damage, encompassing both sporadic CAA and hereditary Dutch-type CAA (D-CAA). The objective is to provide a comprehensive viewpoint to steer future research and potential therapeutic approaches. Delving into the neuroinflammatory reaction to Aβ within the cerebrovasculature may yield insights into CAA's pathogenesis and the occurrence of ARIA.

## ARIA and CAA-ri

Some patients with CAA develop a strong immune response to vascular Aβ in the form of CAA-ri. While estimates suggest an occurrence of approximately 0.13 cases per 100,000 individuals,^7^ CAA-ri is likely underreported. Over the past decades, various descriptions of this clinical condition have emerged. In ‘amyloid beta-related angiitis' (ABRA), there is histopathological evidence of vasculitis, granulomatous inflammation, and meningeal lymphocytosis. The term CAA-ri was introduced later, for a syndrome characterized by radiological signs of CAA paired with vasogenic oedema, responsive to immunosuppressive therapy.^5^ ABRA is specifically reserved for patients demonstrating angiodestructive vasculitis upon histopathological examination. The variability in descriptions suggests that CAA-ri represents a spectrum where the severity and extent of the inflammatory reaction may differ.

Symptoms of CAA-ri range from subacute encephalopathy and seizures to headaches and focal neurological deficits.^8^ MRI white matter hyperintensities, that are typically asymmetrical and indicative of vasogenic oedema, as well as cortical and subcortical haemorrhagic lesions are typically seen in patients with CAA-ri and can be used to diagnose patients without brain biopsy.^8^ Of note, regions of vasogenic oedema in CAA-ri typically co-localize with cerebral microbleeds,^9^ suggesting an association between vessel rupture and local inflammation. CAA-ri may also reflect inflammatory-mediated Aβ clearance. In a CAA-ri case who underwent PET scans before and after the onset of CAA-ri, showed a localized reduction in Aβ deposition in the posterior medial frontal lobe, consistent with the area of vasogenic oedema.^10^ Although it's important to note that these changes in amyloid PET tracer uptake were minimal and should be interpreted with caution, this observation could suggests that the inflammatory response may facilitate the removal of Aβ. Similar PET findings in two patients with CAA-ri also support this hypothesis.^11^ Among patients with CAA-ri, Aβ-autoantibodies have been described, potentially leading to Aβ removal and causing oedema and haemorrhages,^12^ although these Aβ-autoantibodies have not yet been replicated. Previous studies on brain tissue samples from patients with CAA-ri revealed the accumulation of mononuclear and multinucleated white blood cells around affected blood vessels, primarily of the monocyte/microglial lineage. T lymphocytes, including CD8-positive and CD4-positive subsets, were also observed in small numbers, while B lymphocytes were absent.^13^ Notably, published cases often exhibit the apolipoprotein E ε4/ε4 genotype, estimated at 34%, which is significantly higher than the general population. ApoE ε4 may play a role in this context, being not only a significant risk factor for non-familial AD and CAA, but also influencing Aβ deposition and neuro-inflammation.^14^

CAA-ri shows similarities with ARIA, a side effect observed in individuals receiving anti-amyloid immunotherapy for AD. Individuals treated with anti-amyloid monoclonal antibodies are at risk for ARIA, which can be divided into two types. ARIA-E (oedema), seen as leakage of proteinaceous fluid, are identified by hyperintensities on fluid attenuation inversion recovery (FLAIR) MRI, indicative of vasogenic oedema and sulcal effusions with occasional enlargement of the gyri. ARIA-H (haemorrhage), caused by leakage of blood is marked by parenchymal microbleeds and the accumulation of superficial hemosiderin in the leptomeninges, visible on T2∗-weighted MRI scans.^2^ The presence of CAA is known to be an important risk factor for ARIA, and notably recent clinical trials have not included patients with more than four microbleeds in an effort to minimize ARIA risk. As in CAA-ri, ARIA-H frequently co-localizes with regions of ARIA-E. It remains uncertain whether ARIA is mainly an inflammatory reaction associated with the migration of Aβ from parenchymal plaques to the perivascular spaces, direct elimination of Aβ from leptomeningeal and cortical arterioles leading to temporary ‘leaky vessels’, or a combination of both mechanisms.^2^^,^^15^ What is clear, is that ARIA's risk is associated with CAA, the APOEe4 allele, and the dose of the immunotherapy.^15^ Histopathological analysis of ARIA cases has revealed a phagocytic reaction at the location of the Aβ-accumulated vessels, characterized by perivascular inflammation containing macrophages and/or activated microglia. Relative to CAA-ri, cases with ARIA seem to more often show evidence of vasculitis.^16^^,^^17^ While often asymptomatic, ARIA can be fatal, highlighting the urgency to understand the molecular dynamics between inflammation and CAA.^17^

## Neuroinflammation and the pathophysiology of CAA

**Fig. 1:**
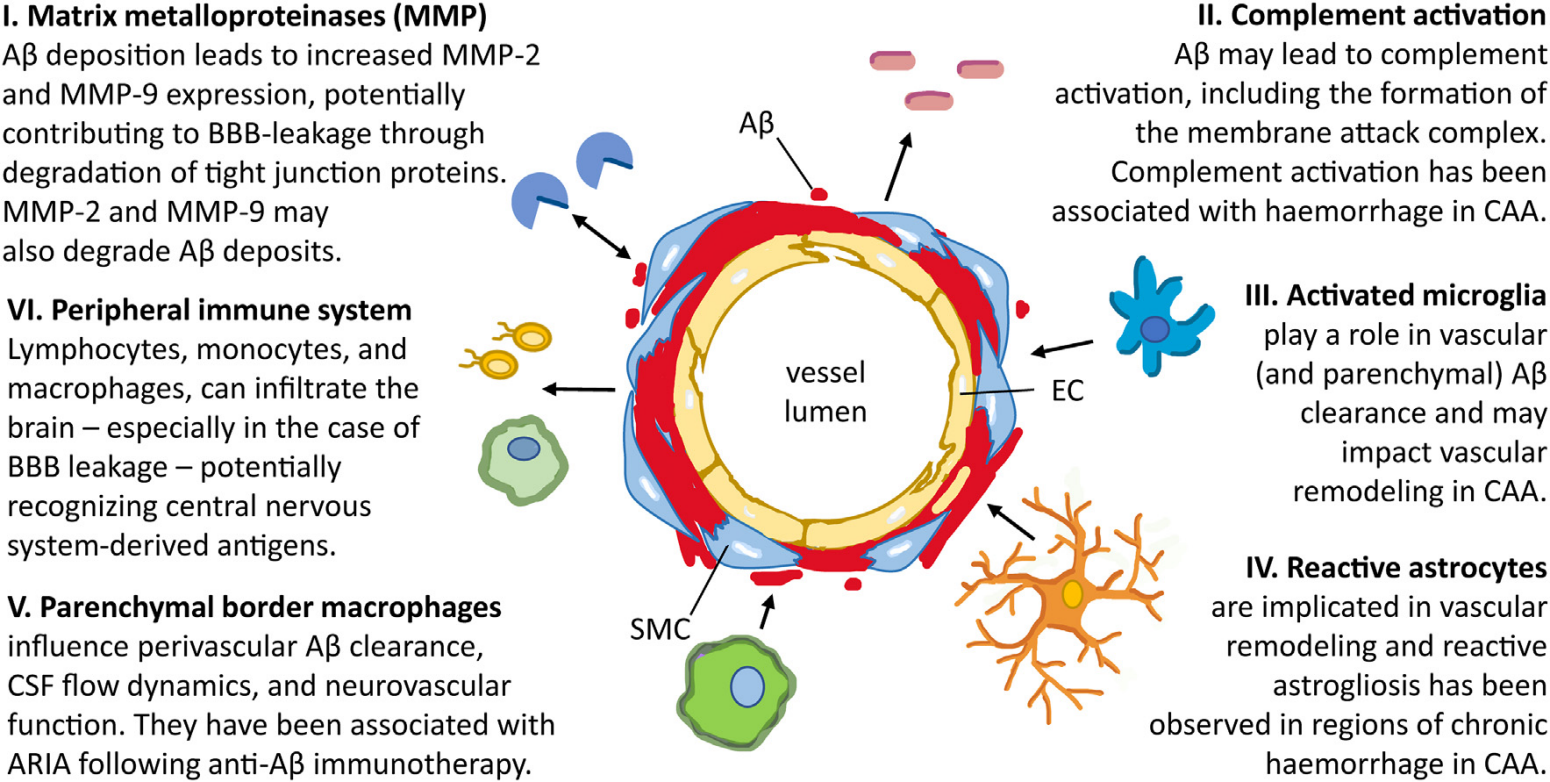
Components of the immune system that have shown interaction with vessels affected by CAA. Aβ, Amyloid-beta; EC, endothelial cell; SMC, smooth muscle cell.

Multiple components of the immune system have been associated with vascular Aβ in CAA. In this section we address the most strongly implicated candidates; also visually represented in Fig. 1. A summary of the studies investigating the relationship between neuroinflammation and CAA is shown in Table 1.

**Table 1:** Overview of evidence for a role of components of neuroinflammation in CAA.

	In vitro studies	Animal studies	Postmortem human brain tissue studies	In vivo human studies
Matrix metalloproteinases	- Exposing endothelial cells to Aβ40 increases MMP-2 and MMP-9 expression and cell death.^18^, ^19^ - MMP-2 and MMP-9 could help degrade Aβ40 and Aβ42.^20^, ^21^	- Aβ increases the expression of MMP-2 and MMP-9 in CAA rat and mouse model.^22^ - Treatment with minocycline inhibits MMP-2 and MMP-9 activity and reduced ICH frequency in CAA mouse models.^23^	- Overexpression of MMP-2 and MMP-9 is observed.^24^, ^25^, ^26^ - MMP expression colocalizes with cerebral microbleeds and vascular Aβ deposits.^24^, ^27^	–
Complement system	- Aβ activates the classical and alternative pathway of the complement system.^28^	- Complement activation is associated with blood–brain barrier injury in CAA mouse model.^29^ - Complement activation is seen in ARIA in CAA mouse model.^30^	- Complement activation colocalizes with vascular Aβ.^31^, ^32^, ^33^ - Expression of components of the MAC complex are associated with ICH and cSS.^32^	–
Activated Microglia	–	- Microglial activation is seen in CAA animal models, and colocalizes with vascular Aβ deposits.^34^, ^35^ - Microglial activation could play a role in vascular Aβ clearance, as observed in CAA mouse models.^36^^,^^37^	- Microglial activation colocalizes with vascular Aβ deposits.^6^^,^^38^ - Microglial activation is observed in remodeled arterioles^6^ and around cerebral microbleeds.^39^ - Microglial function is increased in CAA-ri and ARIA.^17^^,^^40^	–
Reactive astrocytes	–	- Reactive astrocytes are associated with vascular Aβ deposits in CAA animal models.^35^^,^^41^ - Increased cerebral microbleeds are correlated with reactive astrogliosis surrounding vascular Aβ deposits in AD mouse model.^42^	- Reactive astrocytes are observed around remodeled arterioles.^6^ - Reactive astrocytes are seen around cortical iron deposits caused by cSS.^43^ - Reactive astrocytes may play a role in degrading Aβ.^44^	- GFAP in CSF and serum are increased in patients with advanced CAA.^45^
Parenchymal border macrophages	–	- Perivascular macrophages are associated with vascular Aβ deposition in AD mouse model.^46^ - Parenchymal border macrophages may play a role in CSF flow and Aβ clearance in AD mouse model.^47^ - Parenchymal border macrophages may play a role in neurovascular dysfunction in CAA mouse model.^48^^,^^49^ - Perivascular macrophages are associated with ARIA in AD mouse model.^50^	–	–
The peripheral immune system	–	–	–	- Systemic inflammation might be associated with vascular injury in areas commonly affected by vascular Aβ.^51^ - Systemic inflammation has been linked to ICH.^52^^,^^53^ - Patients with CAA-ri often have other systemic autoimmune diseases and autoimmunity may play a role in CAA-ri.^54^, ^55^, ^56^, ^57^, ^58^

### Matrix metalloproteinases

Matrix metalloproteinases (MMPs) are enzymes that play an important role in regulating the remodelling and breakdown of the extracellular matrix. Two specific MMPs; MMP-2 and MMP-9, are of particular interest in the context of CAA, because these are expressed in smooth muscle cells and endothelial cells in blood vessels.^59^ To maintain tissue homeostasis, MMP activity is tightly regulated by controlling activation of pro-MMPs, transcription, and inhibition of active forms by specific tissue inhibitors of MMPs (TIMPs). MMP dysregulation that leads to increased MMP activity can cause excessive tissue degradation and has been implicated in leakage of the blood–brain barrier.^60^ When the blood–brain barrier is compromised, proteins can leak into the brain including fibrinogen, which undergoes conversion into fibrin. Blood–brain barrier leakage is known to trigger inflammation, and recent work suggests that fibrin scaffolds themselves can facilitate immune cell adhesion and activation, leading to chronic inflammation and subsequent neurodegeneration.^27^

MMP dysregulation has been implicated in CAA-related haemorrhage. Postmortem human brain tissue of patients with CAA has shown increased MMP-2 and MMP-9 expression.^24^^,^^25^^,^^26^ This expression was more prominent in the hemisphere with ICH than in the contralateral hemisphere^25^ and expression was found to colocalize with cerebral microbleeds and vascular amyloid deposition.^24^^,^^25^ When comparing expression of MMPs and TIMPs in brain tissue from patients with CAA with ICH versus without ICH, MMP-9 was higher and TIMP3 was lower in CAA cases without a history of ICH, suggesting that a disbalance in MMP expression and inhibition could indeed be associated with CAA-related ICH.^26^

Aβ deposition may play a direct role in MMP dysregulation. Exposing both animal and human endothelial cells to Aβ40 in in vitro models, led to higher expression of MMP-2 and MMP-9, which also resulted in increased cell death.^18^^,^^19^ Evidence from animal models shows that Aβ increases the expression of MMP-2 and MMP-9 and reduces the expression of tight junction proteins (claudin-1 and claudin-5) in ex vivo rat brain microvessels and in vivo brain microvessels of Tg2576 transgenic mice, a mouse model of cerebral amyloidosis.^22^ Together this could contribute to the loss of blood–brain barrier integrity and potentially vessel rupture and haemorrhage. Supporting evidence for this hypothesis comes from a treatment study in two CAA mouse models (Tg2576 and 5xFAD/ApoE4 knock-in) where treatment with minocycline which, among other actions, inhibits MMP-2 and MMP-9 activity, was shown to reduce ICH frequency.^23^

Of note, there is also some evidence to suggest that MMP-2 and MMP-9 might play a role in Aβ degradation. From in vitro studies in human endothelial cells, it seems that MMP-2 and MMP-9 can help degrade Aβ40 and Aβ42 into Aβ16, a non-toxic degradation product,^20^^,^^21^ with Dutch type Aβ40 being more resistant to degradation than the sporadic type Aβ40.^21^ Taken together, the available literature emphasizes the complexity of the mechanisms elicited by MMPs.

### Complement system

The complement system is a vital component of the innate immune system. It is a complex cascade of protein cleavages and interactions that can be triggered through three pathways: the classical pathway, initiated by antibody-antigen binding; the lectin pathway, initiated by mannose-binding lectin binding to pathogen surfaces; and the alternative pathway, which can be activated by spontaneous C3 hydrolysis or foreign surface interaction.^61^ Independent from the triggering method, the cascade progresses similarly. It facilitates pathogen recognition by immune cells, orchestrates immune cell recruitment through signalling, and leads to the formation of the membrane attack complex (MAC).^61^ MAC creates pores in the membranes of target cells, inducing cell lysis. The MAC is of interest in CAA given that it has the potential to play a role in vessel rupture.

Postmortem brain tissue from patients with CAA has shown colocalization of vascular Aβ and complement activation,^32^^,^^33^ with higher complement positivity as the severity of vascular Aβ increases.^31^ Importantly, C5b-9 and C6 expression (both components of the MAC) in arteries/arterioles was associated with subcortical haemorrhage and cortical superficial siderosis.^32^ These findings suggests that vascular Aβ, through initiation of the complement system, could cause the formation of MAC in Aβ laden vessels, possibly resulting in vessel rupture. Indeed, there is evidence that Aβ can directly activate both the classical pathway and the alternative pathway of the complement system.^28^ There is no clear answer to the question how Aβ activation of the complement cascade results in the formation of MAC in the Aβ laden vessels specifically, but there are proposed mechanisms. A recent study by Hu et al. (2023) using skin samples from patients with CAA and brain tissue from the Tg-SwDI/B transgenic mouse model, suggests that migrasomes (extracellular vesicles that are produced during cellular migration) derived from Aβ-stimulated macrophages could play a role.^29^ These migrasomes were found to specifically stick to endothelial cells and are packed with complement activation associated molecules, which are associated with blood–brain barrier injury. Interestingly, complement inhibitory treatment in the transgenic mice could protect against this blood–brain barrier injury.^29^ Another mechanism was proposed by Zabel et al. (2012). This study used post-mortem brains of patients with AD with and without concomitant CAA pathology and found that C3b of the complement cascade is a high affinity ligand for Aβ and that this C3b/Aβ complex can again be bound by microglia expressed CD11b. The authors hypothesize that the CD11b that is bound by the C3b/Aβ complex is delivered to the blood vessels, which leads to vascular Aβ deposition and propagation of MAC in the vessel wall.^33^ Recent work also suggests that these proposed complement-mediated mechanisms could play a role in the pathogenesis of ARIA, further supporting the role of complement in CAA-related vascular damage.^30^

### Activated microglia

Microglia are part of the innate immune system and are the resident phagocytic cells of the central nervous system.^62^ They constantly monitor their environment for abnormalities and foreign infiltrates and can be triggered to activate by various types of danger signals. Aβ is a known trigger for microglial activation.^62^

Activated microglia are observed in the context of vascular Aβ deposition, both in mouse models as well as in human postmortem brain tissue. There is a clear and large microglial response in animal models of CAA,^34^^,^^35^ with a strong spatial correlation between the activated microglia and vascular Aβ deposits.^34^^,^^35^ This co-localization is also reported in studies in postmortem brain tissue of CAA and D-CAA cases.^6^^,^^38^ More specifically, activated microglia are particularly found in later disease stages, for example surrounding arterioles that show evidence of vascular remodelling^6^ as well as in association with perihematomal cerebral microbleeds.^39^

Microglia activation has been implicated to play a role in vascular Aβ clearance. A study that crossed the 5xFAD mouse model of CAA with FIRE mice to create a mouse model that was entirely microglia deficient, observed that his microglial deficiency resulted in abundant vascular Aβ deposition.^36^ Conversely, a study that treated the Tg-SwDI mouse model of CAA with a TLR9 agonist to activate microglia, found significant reduction of vascular Aβ load.^37^ As mentioned in the complement section above, Zabel et al. (2012) propose a mechanism where microglia, through binding to CD11b, deliver a C3b/Aβ-complex to the blood vessels for clearance of Aβ.^33^ A possible role for TREM2 should also be mentioned. TREM2 is mostly expressed by microglia and has been implicated to play a protective role in AD pathogenesis due to its anti-inflammatory and phagocytic properties.^63^ Because of this, microglial function may be an important therapeutic target in AD. If TREM2 could have a similar protective role in CAA is not clear. Importantly, however, microglial function has been linked to ARIA pathogenesis. There is evidence of increased microglial function in patients with CAA-ri and ARIA after anti-Aβ treatment^17^^,^^40^ and ARIA has been observed after TREM2 activating treatment in the ongoing INVOKE-2 clinical trial.^30^ These contrasting observations indicate that it is unclear whether microglial activation is generally good or bad, and that future therapeutic targets will likely need to target specific microglial actions.

### Reactive astrocytes

Reactive astrocytes are a subtype of astrocytes that undergo a series of changes in response to pathological situations in their surrounding tissue.^64^ Although imperfect, a distinction is often made between A1/pro-inflammatory and A2/anti-inflammatory astrocytes. Hypertrophy of reactive astrocytes has long been recognized as a sign of pathology in the central nervous system,^64^ and also in CAA.

Vascular Aβ has been associated with reactive astrogliosis in various animal models of CAA.^35^^,^^41^ More precisely, astrocytic reactivity is seen in response to CAA pathology and more so with increasing deposition of vascular Aβ.^35^^,^^41^ A study in Tg-FDD transgenic mice observed a particularly strong presence of the A1/pro-inflammatory type astrocytes, which decreased TREM-2 protein levels in microglia and thus directly affected microglial homeostasis as well.^41^

There is evidence pointing towards a role for reactive astrocytes in vessel remodelling and haemorrhage in CAA. A study that treated 5xFAD mice with anti-Aβ immunotherapy showed increased cerebral microbleeds severity was highly correlated with reactive astrogliosis surrounding CAA pathology.^42^ Based on observations in postmortem human brain tissue, it also seems that reactive astrocytes are mostly apparent in the immediate perivascular area of CAA affected arterioles that show evidence of vascular remodelling.^6^ Also, a recent study in human brain tissue found that there was a strong local relation between cortical iron deposits, observed in regions of cortical superficial siderosis, and reactive astrocytes.^43^ As cortical superficial siderosis is one of the most important predictors of intracerebral haemorrhage, this finding may suggest that astrogliosis could play a role in vessel rupture. Additionally, from studies in AD it seems that reactive astrocytes could play a role in phagocytizing Aβ,^44^ although such observations have not yet been reported for vascular Aβ.

Glial fibrillary acidic protein (GFAP) is the most used marker to identify reactive astrocytes in tissue,^64^ and recent studies suggest that GFAP can be measured in vivo in CSF, plasma and serum. Whether such measurements could be sensitive and specific enough to serve as a biomarker for CAA still needs to be determined. A first study measuring plasma GFAP concluded that plasma GFAP levels do not represent vascular Aβ pathology.^65^ However, a recent study found that GFAP levels in CSF and serum were increased in patients with advanced CAA compared to controls and that in D-CAA GFAP levels in CSF were increased years before symptom onset.^45^

### Parenchymal border macrophages

Central nervous system border-associated macrophages are macrophages that are part of the innate immune system that reside at the borders of the central nervous system, such as in the perivascular space and around the leptomeninges, choroid plexus, and dura.^66^ Of particular interest in CAA are parenchymal border macrophages (i.e. leptomeningeal and perivascular macrophages), because of their location in close proximity to vascular Aβ deposition.

There is evidence suggesting that perivascular macrophages play a role in regulating CAA pathology. A study in the TgCRND8 mouse model of AD found that depletion and stimulation of perivascular macrophages resulted in increased and decreased CAA severity respectively, which was not mediated by activated microglia or reactive astrocytes.^46^ This role in regulating CAA severity may be in part mediated by their role in CSF flow dynamics. A recent study in mice showed that parenchymal border macrophages play a role in regulating arterial motion and extracellular matrix remodelling, which both affect CSF flow.^47^ Notably, when parenchymal border macrophages were depleted in the 5xFAD mouse model of AD, CSF flow was impaired and a significantly increased parenchymal and vascular Aβ plaque load was observed after 1-month compared to the non-depleted 5xFAD mice.^47^ These findings indicate that parenchymal border macrophages may play a role in perivascular Aβ clearance from the brain.

Parenchymal border macrophages have, however, also been implicated to play a role in the neurovascular dysfunction that Aβ induces in the brain. Studies in Tg2576 mice have shown that parenchymal border macrophages are enriched in CD36, which underlies the oxidative stress and neurovascular dysfunction induced by Aβ.^48^^,^^49^ Deletion of CD36 rescued neurovascular function in this mouse model,^48^^,^^49^ reduced Aβ1-40 accumulation, and improved clearance of exogenous injected Aβ1-40, which in one study resulted in improved cognition.^49^

Finally, perivascular macrophages have been associated with ARIA after anti-Aβ immunotherapy. A study in an AD mouse model, PDAPP hTau APP KI, found that cerebral microbleeds that are seen after anti-Aβ immunotherapy are associated with the local activation of perivascular macrophages associated with vascular Aβ deposits.^50^ This perivascular macrophage activation was coupled with an increased expression of inflammatory signalling and of matrix remodelling genes. The authors concluded that this enhances vascular permeability and susceptibility to cerebral microbleeds.^50^

### The peripheral immune system

The peripheral immune system is increasingly recognized as both a contributor to and responder to the damage caused by CAA. Systemic inflammation plays a pivotal role in influencing the progression of CAA by affecting vascular health and coagulation.

Peripheral immune cells, including lymphocytes, monocytes, and macrophages, can infiltrate the brain, potentially recognizing central nervous system-derived antigens as foreign and initiating an immune response. Several studies have demonstrated that systemic inflammation is preferentially associated with vascular injury in areas commonly affected by vascular Aβ pathology.^51^ Moreover, systemic inflammatory events such as flu or fever^52^ and presence of dental caries and periodontal disease^53^ have been linked to the onset of intracerebral haemorrhages, highlighting the connection between systemic inflammation and brain vessels.

Patients with CAA-ri often have other systemic autoimmune diseases (such as hypothyroidism, Grave's disease, and rheumatoid arthritis) and autoimmunity may play a role in the pathogenesis of CAA-ri, particularly in genetically susceptible individuals, as environmental triggers can lead to immune dysregulation.^54^ This hypothesis is further supported by case reports of patients developing CAA-ri while receiving systemic immunotherapy such as anti-programmed death-1 (PD-1) monoclonal antibodies,^55^ tyrosine kinase inhibitor,^56^ chemotherapy^57^ or monoclonal antibodies for cancer treatment.^58^ Notably, individuals carrying the ApoE ε4 allele exhibit heightened systemic inflammatory responses to intravenous LPS stimulation,^4^ potentially explaining the association with developing CAA-ri.

## Outstanding questions

While the described literature points towards an important role for neuroinflammation in CAA, the key question remains whether targeting the immune response could serve as a potential treatment. Immunosuppression has been proven beneficial for patients with CAA-ri, but it is uncertain whether targeting inflammatory pathways might help the whole CAA patient population, including patients who do not exhibit clear clinical signs of inflammation. Given the likely role of inflammation in vessel remodelling and possibly in haemorrhage in CAA, reducing inflammation surrounding Aβ laden vessels, while maintaining homeostasis, could potentially reduce vascular damage in many more patients with CAA. Here we discuss some potential treatment targets under investigation.

## Potential treatment targets

One of the most promising targets to date has been the inhibition of MMPs. In animal models, MMP inhibitors demonstrate efficacy in reducing MMP activation and oxidative stress, albeit without impacting CAA progression.^67^ Furthermore, minocycline treatment (known to have MMP inhibitory properties) in mice significantly decreases haemorrhage frequency and inflammatory markers in the brain without affecting vascular Aβ load.^23^ In a small group of patients with clinically aggressive CAA, minocycline appeared safe and generally tolerated and was associated with reduced haemorrhage occurrence.^68^ However, in a recent study of patients with moderate to severe non-CAA cerebral small vessel disease, minocycline treatment showed no correlation with changes in microglial signals as measured by PET-imaging and serum inflammatory markers were unaffected.^69^ To assess target engagement in a CAA patient population, the recently completed randomised controlled BATMAN trial aims to assess the effect of minocycline on markers of neuroinflammation (IL-6, MCP-1 and IBA-1) and the gelatinase pathway (MMP2/9 and VEGF) in CSF in patients with sporadic CAA and D-CAA.^70^

Another potential treatment option is to administer antioxidants, as they support the immune system by protecting cells from oxidative stress and damage caused by free radicals. Uric acid, which acts as an antioxidant, has been shown to reduce the generation and aggregation of Aβ and to maintain the integrity of vascular walls in APP23 mice.^71^ Similarly, taxifolin, a plant-based antioxidant, has been reported to reduce Aβ aggregation in vitro.^72^

While effective in the treatment of CAA-ri, the potential for corticosteroids to mitigate long-term vascular damage in CAA remains unclear. A study on cultured human cerebrovascular smooth muscle cells showed that although dexamethasone did not significantly affect the buildup of fibrillar Aβ on cell surfaces, it effectively reduced IL-6 levels and MMP-2 activation.^19^ This suggests that dexamethasone could potentially reduce inflammation and long-term weakening of vessel wall integrity.

The effects of long-term immunosuppression are, however, not known. Apart from the unknown efficacy, it is important to state that the immune system plays a crucial role in maintaining brain homeostasis, and neuroinflammation is not merely bad. General immunosuppression may leave patients immunocompromised which is not without risks and may therefore not be a feasible long-term treatment option. We saw, for example, that the immune response is also involved in the clearance of vascular Aβ from the brain. It therefore seems likely that more specific immunosuppression might be more effective and safer for long-term treatment. To this end, other treatment considerations could be to target specific components (e.g. C1, C3, C5) of the complement cascade to halt the MAC formation.^30^ Such an approach is currently being tested in the context of preventing secondary brain injury from aneurysmal subarachnoid haemorrhage.^73^ Another target could be the development of therapeutics that target very specific cell types, such as perivascular macrophages or A1/pro-inflammatory astrocytes. In order to find the best and most specific targets, a better understanding of the dynamic influences of the immune system across different stages of CAA pathology is needed. Finding out which components of immune activation play the most direct role in vessel remodelling and haemorrhage will be key. Possible approaches for future research could be to use omics methods to explore the precise inflammatory profile around CAA laden vessels. This approach could help identify mechanistic targets. Those can then be tested in animal models, for which the appropriate animal models that display the inflammatory features will need to be established, and in human studies, for example by testing already established therapeutics that target specific components of the immune system.

Recognizing the overlap between CAA and AD underscores the importance of enhancing vascular function to address both conditions. Successful treatment strategies in CAA-ri may also hold promise for preventing ARIA in patients receiving amyloid reducing immunotherapies for AD. Conversely, insights from studies on inflammation in AD may also benefit the CAA population. For instance, treatments targeting glial senescence with senolytics hold promise for yielding positive outcomes in individuals with CAA.^74^ Besides, fibrin-targeting immunotherapy has shown potential in reducing autoimmunity- and amyloid-driven neurotoxicity, without suppressing innate immunity or affecting coagulation in animal models for AD.^75^ These findings underscore the potential for interdisciplinary approaches to address the complex pathophysiology of CAA and related conditions, paving the way for more effective therapeutic strategies in the future.

## Conclusion

This review of studies examining neuroinflammation in CAA reveals a multifaceted interplay between Aβ, vascular function, and the immune system. Data from animal models and histopathological studies to date suggest that: 1) Aβ may lead to MMP dysregulation and cause leakage of the blood–brain barrier; 2) complement activation shows colocalization with Aβ and has the potential to result in vessel rupture; 3) activated microglia and reactive astrocytes show a spatial correlation with Aβ and might be involved in vessel remodelling; 4) parenchymal border macrophages are involved in regulating CAA severity through their role in CSF flow dynamics and mediate the influence of Aβ on neurovascular function; 5) systemic inflammation likely influences vascular damage in the brain. Understanding the dynamic influence of the immune system across different stages of CAA pathology should be a focus in future studies, as this may lead to the identification of novel, more specific, immunosuppressing targets.

## Contributors

HvdB, MK and EvE drafted the manuscript. SV performed the literature search and provided critical revisions. EvE drafted the figure. All authors read and approved the final version of the manuscript.

## Declaration of interests

MK has received consulting fees from Kisbee Therapeutics and Hoffman–La Roche, and receives research funding through a sponsored research agreement with Therini Bio. EvE has received reimbursement for travel expenses and accommodation costs for the international CAA conference. She is an unpaid member of the steering committee for a multicenter CAA treatment trial by Alnylam Pharmaceuticals Inc. She is also part of a workgroup on ARIA in collaboration with the American Alzheimer's Association (unpaid). HvdB and SV declare no conflict of interest.
